# P2Y_2_ receptor modulates shear stress-induced cell alignment and actin stress fibers in human umbilical vein endothelial cells

**DOI:** 10.1007/s00018-016-2365-0

**Published:** 2016-09-20

**Authors:** Ramasri Sathanoori, Paulina Bryl-Gorecka, Christa E. Müller, Laurie Erb, Gary A. Weisman, Björn Olde, David Erlinge

**Affiliations:** 1 Department of Cardiology, Clinical Sciences, Lund University, Tornavägen 10, BMC-D12, Room D1219b, 221 84 Lund, Sweden; 2 PharmaCenter, Pharmaceutical Institute, Pharmaceutical Chemistry I, University of Bonn, Bonn, Germany; 3 Department of Biochemistry, University of Missouri, Columbia, USA

**Keywords:** Shear stress, P2Y_2_ receptors, Integrins, FAK, Wound repair

## Abstract

Endothelial cells release ATP in response to fluid shear stress, which activates purinergic (P2) receptor-mediated signaling molecules including endothelial nitric oxide (eNOS), a regulator of vascular tone. While P2 receptor-mediated signaling in the vasculature is well studied, the role of P2Y_2_ receptors in shear stress-associated endothelial cell alignment, cytoskeletal alterations, and wound repair remains ill defined. To address these aspects, human umbilical vein endothelial cell (HUVEC) monolayers were cultured on gelatin-coated dishes and subjected to a shear stress of 1 Pa. HUVECs exposed to either P2Y_2_ receptor antagonists or siRNA showed impaired fluid shear stress-induced cell alignment, and actin stress fiber formation as early as 6 h. Similarly, when compared to cells expressing the P2Y_2_ Arg-Gly-Asp (RGD) wild-type receptors, HUVECs transiently expressing the P2Y_2_ Arg-Gly-Glu (RGE) mutant receptors showed reduced cell alignment and actin stress fiber formation in response to shear stress as well as to P2Y_2_ receptor agonists in static cultures. Additionally, we observed reduced shear stress-induced phosphorylation of focal adhesion kinase (Y397), and cofilin-1 (S3) with receptor knockdown as well as in cells expressing the P2Y_2_ RGE mutant receptors. Consistent with the role of P2Y_2_ receptors in vasodilation, receptor knockdown and overexpression of P2Y_2_ RGE mutant receptors reduced shear stress-induced phosphorylation of AKT (S473), and eNOS (S1177). Furthermore, in a scratched wound assay, shear stress-induced cell migration was reduced by both pharmacological inhibition and receptor knockdown. Together, our results suggest a novel role for P2Y_2_ receptor in shear stress-induced cytoskeletal alterations in HUVECs.

**Electronic supplementary material:**

The online version of this article (doi:10.1007/s00018-016-2365-0) contains supplementary material, which is available to authorized users.

## Introduction

Mechanical forces such as fluid shear stress directly affect the endothelial cells lining the vessel wall and coordinate the complex events of vasodilation and vascular hemostasis. These hemodynamic forces also play an important role in vascular remodeling, and pathophysiology [1]. In regions of laminar shear stress, endothelial cells elongate and align in the direction of flow in contrast to atheroprone regions of arterial bends and bifurcations where these cells display polygonal or cobblestone morphology, a feature also evident in a murine model [2–4]. The blood flow patterns at these atherogenic regions result in endothelial injury causing dysfunction and eventually contributing to atherosclerosis [1]. Moreover, laminar shear stress is a positive regulator of endothelial cell migration in wound repair, in vitro [5, 6]. The endothelial cells play a critical role in repair following injuries caused due to ischemia, and medical procedures including vein bypass graft [5]. Furthermore, exposure of venous grafts to arterial biomechanical forces is presumed to be an important stimulus for vascular remodeling and venous arterialization with changes in endothelial cell gene expression [2].

The endothelial cells are influenced either directly, by the action of shear stress, or indirectly by alterations in the local concentration of receptor agonists at their surface. Several different membrane proteins including receptor tyrosine kinases, integrins, G protein-coupled receptors (GPCRs), and ligand-gated ion channels that mediate mechanotransduction in the vessel wall are activated in response to shear stress [7, 8]. Many of these proteins link similar downstream signaling molecules regulating primary flow-induced responses, such as eNOS activation, and engaging in signal coordination and crosstalk. It is well documented that endothelial cells release ATP and UTP, in response to various stimuli including hypoxia, vascular injury, and mechanical stimulation, and that these extracellular nucleotides act as paracrine or autocrine mediators via activation of purinergic P2 receptors [9, 10]. Purinergic receptors are classified into P2X ligand-gated ion channel receptors and P2Y G protein-coupled receptors [11] and among these receptors, the P2X4 receptor is implicated in flow-induced calcium flux, vasodilation, and atheroprotective gene expression [12, 13] while the P2Y_2_ receptor is associated with mechanosensitivity in osteoblasts [14, 15]. Both ATP and/or UTP are shown to influence endothelial cell cytoskeletal changes such as actin filament formation and cell motility, associated with activation of integrins and growth factor receptors [16–18].

Studies have described several cellular components with potential mechanosignaling properties including integrins [19] that associate with focal adhesions and regulate cell migration [5, 19, 20]. The Arg-Gly-Asp (RGD) integrin-binding motif of the P2Y_2_ receptor is known to co-localize with α_v_β_3_ and α_v_β_5_ integrins enabling extracellular nucleotides to activate focal adhesion kinases (FAK) and the integrin signaling pathways [17, 21, 22]. Shear stress increases actin stress fiber (ASF) formation inducing the realignment of focal adhesions, which is related to spatial and temporal responses of the associated proteins including FAK [23, 24]. In fact, shear stress and extracellular nucleotides are shown to induce the phosphorylation of FAK at Tyr397 in endothelial cells [16, 25]. Recently, a study showed that the endothelial P2Y_2_ receptors and associated G_q_/_11_ proteins play a critical role in fluid shear stress-induced nitric oxide (NO) formation and the regulation of vascular tone and blood pressure [26]. However, studies involving shear stress-mediated P2Y_2_ receptor signaling of cytoskeletal assembly and migration in endothelial cells are limited. Here, we investigated the role of these receptors in modulating cytoskeletal changes in response to shear stress and report a novel role for this receptor in shear stress-mediated cell alignment, ASF formation, and wound closure in human umbilical vein endothelial cells (HUVECs).

## Materials and methods

### Reagents

Medium 200 supplemented with basic fibroblast growth factor/heparin, hydrocortisone, human epidermal growth factor, 10 % fetal bovine serum (FBS), and antibiotics; 1X attachment factor was obtained from Life Technologies, USA. QIAzol lysis reagent, miRNeasy^®^ mini kit and RNase free DNase set were from Qiagen, USA. High capacity cDNA reverse transcription kit; TaqMan^®^ assay and master mixes; Lipofectamine^®^ RNAiMAX transfection reagent, OPTI-MEM, ProLong^®^ gold antifade mounting medium, and Silencer Select siRNA for P2Y_2_ were from Life Technologies. Transfex was from ATCC; UTP and ATPγS were from Sigma, USA; MRS-2768 was from TOCRIS Bioscience, UK; Cibacron Blue F3GA was from PolySciences, Germany; AR-C118925, the P2Y_2_ receptor-specific antagonist [27] was from University of Bonn, Germany; Phospho-Stop and complete protease inhibitors were from Roche Life Sciences, USA; Micro BCA protein assay kit was from Thermo Scientific, USA; XT 4–12 % Bis–Tris gel was from BioRad, USA. All reagents were used according to manufacturer’s instructions. The following antibodies were used: Anti-phosphorylated FAK (Y397), anti-FAK, anti-phosphorylated cofilin-1 (S3), anti-cofilin-1, anti-phosphorylated eNOS (S1177), anti-eNOS, anti-phosphorylated AKT (S473), anti-AKT, anti-β tubulin, anti-GAPDH, anti-hemagglutinin (HA) primary antibodies (Cell Signaling Technology, USA), anti-P2Y_2_ receptor (Alomone Labs, Israel), and HRP-conjugated anti-rabbit secondary antibodies for Westerns (Thermo Scientific); ActinRed™555, ActinGreen™488 and NucBlue^®^ ReadyProbes, anti-mouse- and anti-rabbit-Alexa Fluor 555-IgG (Life Technologies) as well as the anti-HA primary antibody for immunocytochemistry.

### Cell culture

HUVECs (Life Technologies) were used between passages 1–4. Cells were cultured in Medium200 supplemented with 10 % fetal bovine serum, 3 ng/ml basic fibroblast growth factor, 10 μg/ml heparin, 1 μg/ml hydrocortisone, 10 ng/ml human epidermal growth factor, gentamycin, and amphotericin. Uniform shear stress was achieved using the orbital shaker model and the in vitro fertilization (IVF) dish with HUVECs restricted to the peripheral area similar to previous studies [12, 28]. Briefly, a shear stress of 1 Pa was used that was calculated according to the formula τ_max_ = a√ηρ (2π*f*)^3^, as enumerated in our previous publication [12]. To observe cell alignment, 4 × 10^5^ cells were seeded in IVF dishes coated with 1× attachment factor containing gelatin. Cultures were synchronized for 24 h in 0.5 % FBS media and subsequently switched to regular media prior to shear stress (6 h) experiments in the presence or absence of P2Y_2_ receptor antagonists (10 μmol/l AR-C118925 and 100 μmol/l Cibacron Blue F3GA). To observe ASF formation and assess protein phosphorylation, cells (1.5 or 4 × 10^5^) were synchronized with 0.5 % FBS for 24 h and subjected to either shear stress or receptor agonists (100 μmol/l ATPγS, 100 μmol/l UTP, or 10 μmol/l MRS-2768) in serum-free media.

### siRNA-mediated knockdown

Transient transfections using scrambled and *P2Y*_*2*_-specific siRNA were performed using Lipofectamine RNAiMax as per manufacturer’s instructions and as previously published [12]. At 3 days post-transfection, the cells were either subjected to shear stress or exposed to the receptor agonists (ATPγS, UTP, and MRS-2768) and harvested for protein or processed for immunocytochemistry.

### RNA isolation and reverse transcription-PCR analysis

Cells were harvested and total RNA was isolated using QIAzol after 48 h of transient transfection with the siRNA constructs. RNA purification, cDNA synthesis, and real-time PCR were performed according to manufacturer’s instructions. TaqMan^®^ assays were used to measure mRNA expression of *P2Y*_*2*_, which was normalized to the housekeeping genes *PPIA* and *18S*.

### P2Y_2_ RGD wild-type (WT) and RGE mutant retroviral vector production and transduction

The retroviral stocks were prepared at the vector core facility, Lund University. Briefly, Phoenix cells were transiently transfected in 100 mm dishes. Ten μg of retroviral vectors (P2Y_2_ RGD-pLSXN WT and P2Y_2_ RGE-pLSXN mutant) [22], 6 μg of pcDNA3.MLV gag.pol, and 5 μg of RD114 plasmid were mixed in 500 μl water with 50 μl 2.5 M CaCl_2_, and then 500 μl of 2X HBS was added to this DNA mixture before incubating it at room temperature for 20 min. This was then added dropwise to the cells and incubated for 6–12 h after which the medium was replaced. Supernatants were harvested 36 h to 48 h after transfection, filtered through 0.45 μm filter unit, and the virus was concentrated by ultracentrifugation at 100,000×*g* for 90 min at 4 °C. Viral particles were suspended in normal DMEM and stored at −80 °C. Retroviral titers were determined by limiting dilution with HEK293 cells.

For retroviral expression of P2Y_2_ RGD WT and P2Y_2_ RGE mutant receptors, 2 × 10^5^ HUVECs were seeded in the IVF dishes 24 h before infection in complete growth medium to obtain cultures in the exponential growth phase. On the day of infection, the retroviral particles (~MOI of 10 cfu/cell) were added to medium supplemented with 8 μg/ml polybrene. Cells with medium containing polybrene without the retrovirus served as a control. As there was no apparent toxicity, the cells were incubated for 48 h and were then used for experiments.

### Immunoblot analysis

Immunoblotting was performed using SDS-PAGE by loading equal protein content and thereafter transferred onto nitrocellulose membranes. The membranes were subsequently blocked for 1 h at room temperature in 50 mmol/l Tris–HCl (pH 7.6), 137 mmol/l NaCl, and 0.2 % (w/v) Tween 20 (TBS-T) containing either 5 % (w/v) nonfat dried milk. They were then incubated at 4 °C with the primary antibodies in TBS-T containing 5 % (w/v) protease-free BSA. The bands were visualized by enhanced chemiluminescence using horseradish peroxidase-conjugated secondary antibody and images acquired with LI-COR Odyssey^®^ Fc dual-mode imaging system. Band intensities were quantified using the Image Studio™ software and the phosphorylated protein was normalized to the respective total protein levels.

### Actin staining with phalloidin and immunocytochemistry

Immunofluorescence was performed on cells fixed with ice-cold 4 % paraformaldehyde for 20 min followed by permeabilization with 0.2 % Triton-X 100 in 1X PBS for 15 min at room temperature. Subsequent to washes, the cells were blocked with 5 % normal serum and then incubated in anti-HA primary antibody overnight at 4 °C in a humidified chamber. The following day, the cells were washed and incubated with Alexa fluor 555-conjugated secondary antibody for 2 h at room temperature in the dark. Finally, the cells were stained with fluorescently labeled phalloidin (ActinRed™555 or ActinGreen™488) and nuclei (NucBlue^®^) as per the manufacturer’s protocol. The cells on coverslips were mounted with ProLong^®^ gold antifade and left to cure overnight. Images were visualized in an Olympus BX60 (Olympus, Japan) epifluorescence microscope and acquired using a Nikon DS-2Mv camera (Nikon, Japan). ASF were identified by phalloidin staining of filamentous actin and cells positive for ASF were quantified in a total of ≥300 cells for each condition. The cell orientation in response to shear stress was determined by measuring the angle between a cell’s axis and the direction of flow using the angle tool function in ImageJ (NIH) software [29]. The degree of cell alignment was quantified in ≥300 cells per experimental group and the statistical analysis was performed based on the percentage of cells aligned along the 45° angle. All the quantifications (cell orientation and cells positive for ASF) were performed in randomly selected fields from multiple independent experiments in a blinded manner.

### Endothelial cell migration

Human umbilical vein endothelial cells seeded onto gelatin-coated IVF dishes were allowed to reach confluence. The cells were either transfected with siRNA or exposed to receptor antagonists for 1 h before the assay. The cell monolayer was scratched using a 1000 μl pipette tip to create a straight-lined wound in the direction parallel to the flow. The dishes were then rinsed in media to remove cell debris, replenished with serum-free media and were either subjected to shear stress or kept static for 6 h. Phase contrast images of the same area before and after shear stress were acquired and cell migration was analyzed using ImageJ (NIH) software. For each experimental condition, three wounds were made and two fields per wound were imaged and quantified in a blinded manner. A total of five independent experiments in replicates were performed.

### Statistical analysis

All data are expressed as mean ± standard error (S.E.M.). Statistical analysis (GraphPad Prism software) was performed using unpaired Student’s *t* test when comparing two situations, one-analysis of variance (ANOVA) with Bonferroni correction for multiple comparisons with *P* values ≤0.05 regarded as statistically significant.

## Results

### Role of P2Y_2_ receptor in shear stress-induced endothelial cell alignment

**Fig. 1:**
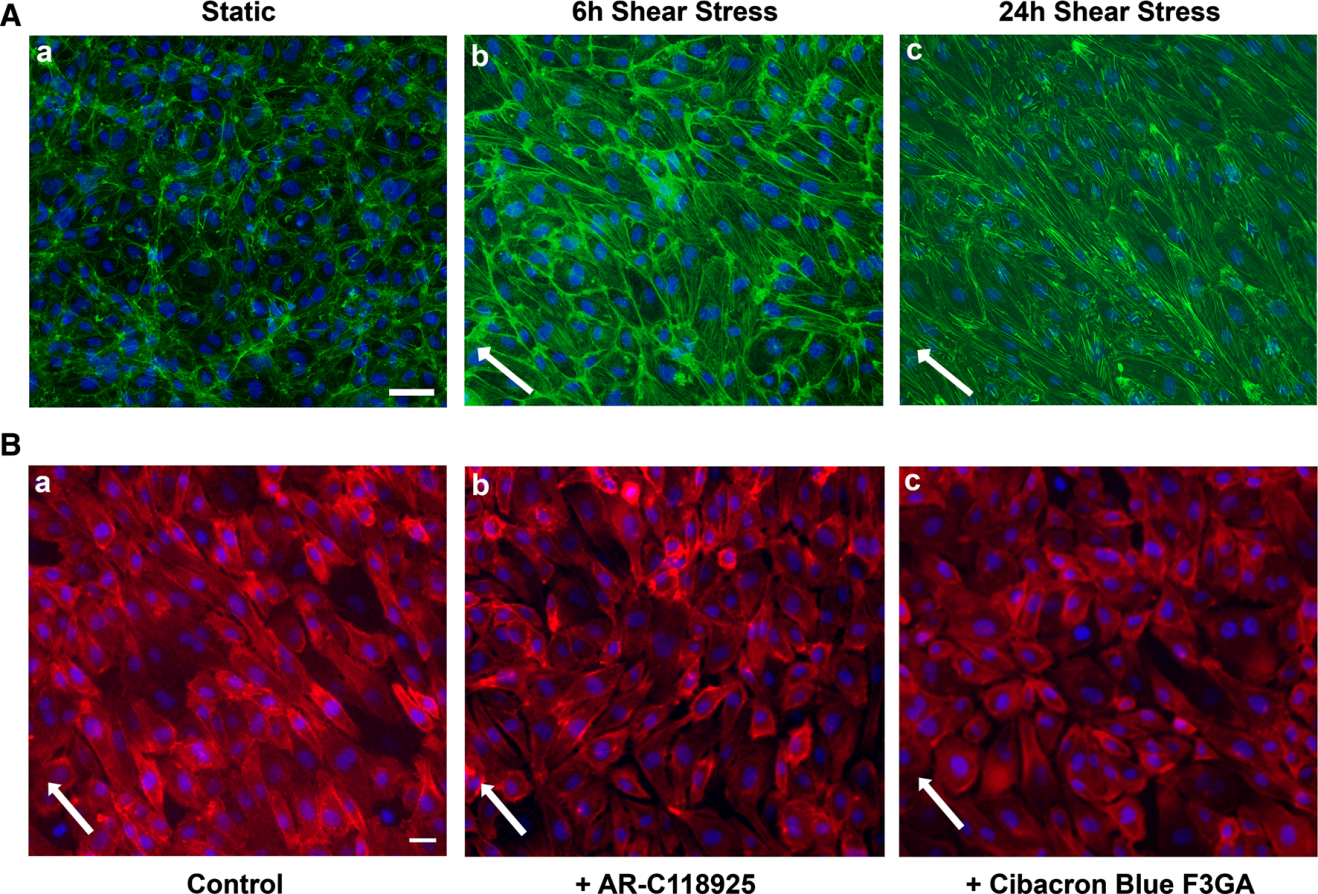
P2Y_2_ receptor antagonists impair shear stress-mediated cell alignment. Cells were subjected to 1 Pa shear stress or kept under static conditions (6 and 24 h) and fixed. To look at the effect of the P2Y_2_ receptor, the monolayers were exposed to the receptor antagonists AR-C118925 and Cibacron Blue F3GA for 1 h and then subjected to shear stress for 6 h and fixed. The epifluorescence images show (×20 objective) representative HUVEC monolayers stained for actin cytoskeleton with ActinGreen™488 (**A**) or ActinRed™555 (**B**) and nucleus with NucBlue^®^. **A** HUVECs under static conditions display cobblestone morphology with no defined orientation (*a*). However, cell elongation and alignment along the direction of flow are observed under shear stress conditions (*b* 6 h and *c* 24 h). (**B**) Representative epifluorescent images (×20 objective) of cells subjected to 6 h shear stress show that compared to control cells (*a*), HUVECs in the presence of the receptor antagonists exhibit impaired alignment (*b*, *c*). *White arrows* indicate the direction of flow. *n* = 4 experiments;* scale bars* are 40 μm (**A**) and 20 μm (**B**)

**Fig. 2:**
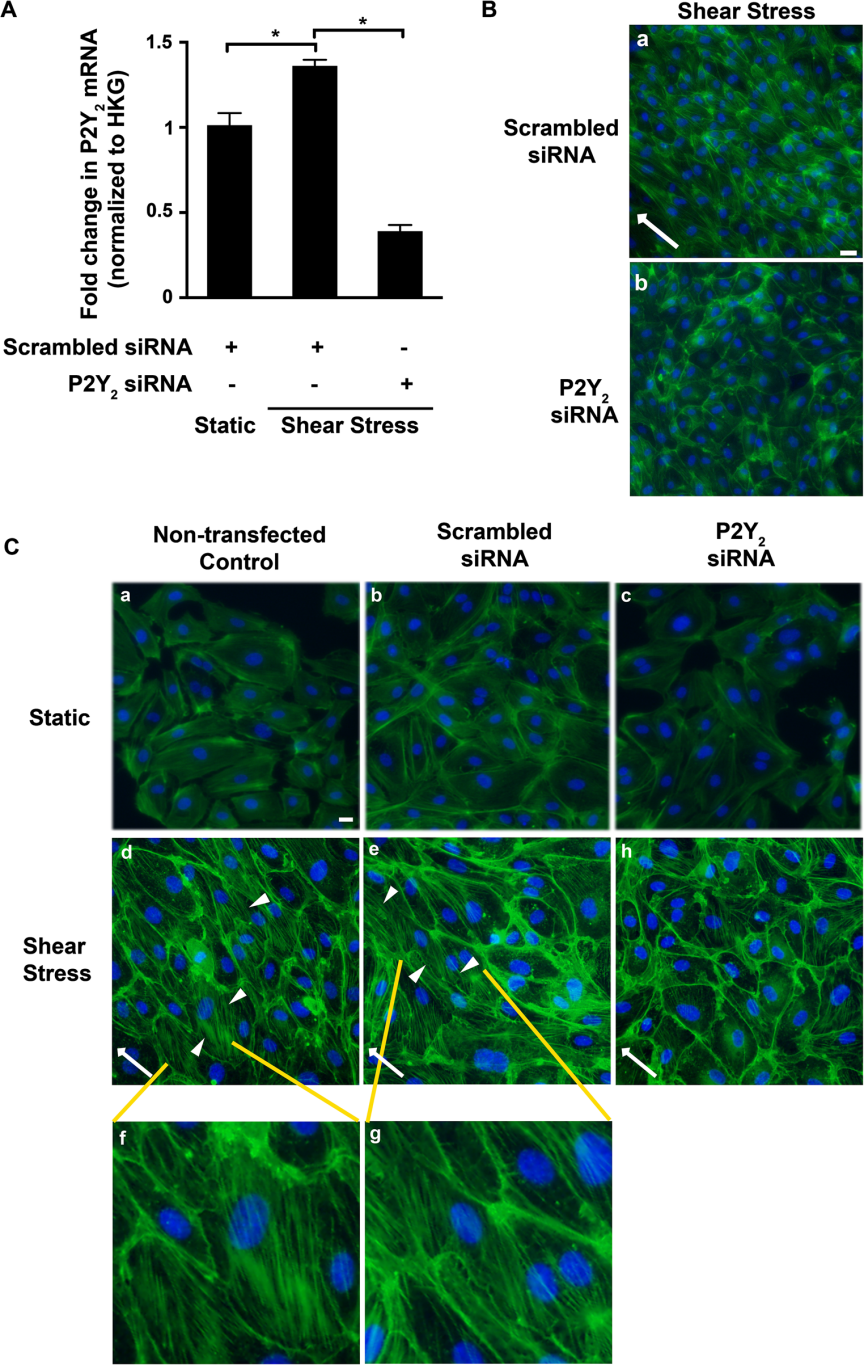
P2Y_2_ receptor knockdown attenuates shear stress-induced cell alignment and actin stress fiber formation in endothelial cells. HUVECs transiently transfected with either scrambled or *P2Y*_*2*_-specific siRNA were subjected to 6 h shear stress or kept under static conditions. Cells were either fixed for immunocytochemistry or processed for RNA extraction. Bar graph (**A**) represents *P2Y*_*2*_ transcript levels under shear stress conditions with or without *P2Y*_*2*_ siRNA knockdown. The mRNA was normalized to the geometric mean of the housekeeping genes (HKG). *n* = 3 experiments; *P ≤ 0.05. Fixed HUVECs were fluorescently labeled with ActinGreen™488 and NucBlue^®^ for actin and the nucleus, respectively. Representative epifluorescence images (×20 objective) of **B** shear stress-induced cell alignment in the direction of flow (*white arrows*) in cells transfected with scrambled siRNA (*a*) but not in cells transfected with *P2Y*_*2*_-specific siRNA (*b*) and **C** phalloidin staining shows the elongated cell shape and induction of numerous ASF (*arrowheads*) oriented in the direction of flow (from *right* to *left*) in both non-transfected controls (*d*) and in cells transfected with scrambled siRNA (*e*) that are magnified in images (*f*) and (*g*), respectively. In contrast, this is not evident in the static cultures (*a*–*c*) and the cells transfected with *P2Y*_*2*_ receptor-specific siRNA (*h*); *n* = 6 experiments; *scale bar* 20 μm

We previously validated the orbital shaker model of shear stress in HUVECs and observed a shear stress-induced increase in *P2Y*_*2*_ mRNA (3.5 ± 0.4-fold; *P* = 0.002) as early as 6 h [12]. Therefore, for the rest of the study, we used 6 h of shear stress to visualize cell alignment. On exposure to 1 Pa shear stress, the endothelial cells were elongated and aligned with their major axis in the direction of flow (45°) and displayed prominent stress fibers along the length of the cells (Fig. 1A). Approximately 60 % of the cells under shear stress oriented at an angle of 45° in contrast to the cells under static conditions that retained cobblestone morphology with random angles of orientation (*P* = 0.00001; Online Resource 1A and B). To test the involvement of the P2Y_2_ receptor in this process, we initially used both the nonselective (100 μmol/l Cibacron Blue F3GA) and the selective (10 μmol/l AR-C118925) antagonists to block the P2Y_2_ receptor. We observe reduced alignment in HUVECs subjected to 6 h of shear stress in the presence of these antagonists (Fig. 1B) with a majority of cells at random angles and only 3 % of the cells aligned in the direction of flow. In comparison, 57.6 % of the cells were aligned in the absence of the P2Y_2_ receptor antagonists (*P* < 0.0001; Online Resource 1C and D). To further confirm the role of the P2Y_2_ receptor, we suppressed receptor expression in HUVECs using the knockdown approach. At 48 h post-transfection, we observe a significant knockdown both in *P2Y*_*2*_ mRNA (71.3 ± 2.7 %; *P* < 0.0001; Fig. 2A) as well as protein (36.5 ± 7.9 %; *P* = 0.03; Online Resource 2). Similar to the effect of the antagonists, we observe reduced cell alignment (Fig. 2B) with 8 % of the cells aligned in the direction of flow with P2Y_2_ receptor-specific siRNA as opposed to 44.8 % in cells transfected with scrambled siRNA (*P* = 0.0002; Online Resource 1E and F).

### P2Y_2_ receptor mediates ASF formation in HUVECs

**Fig. 3:**
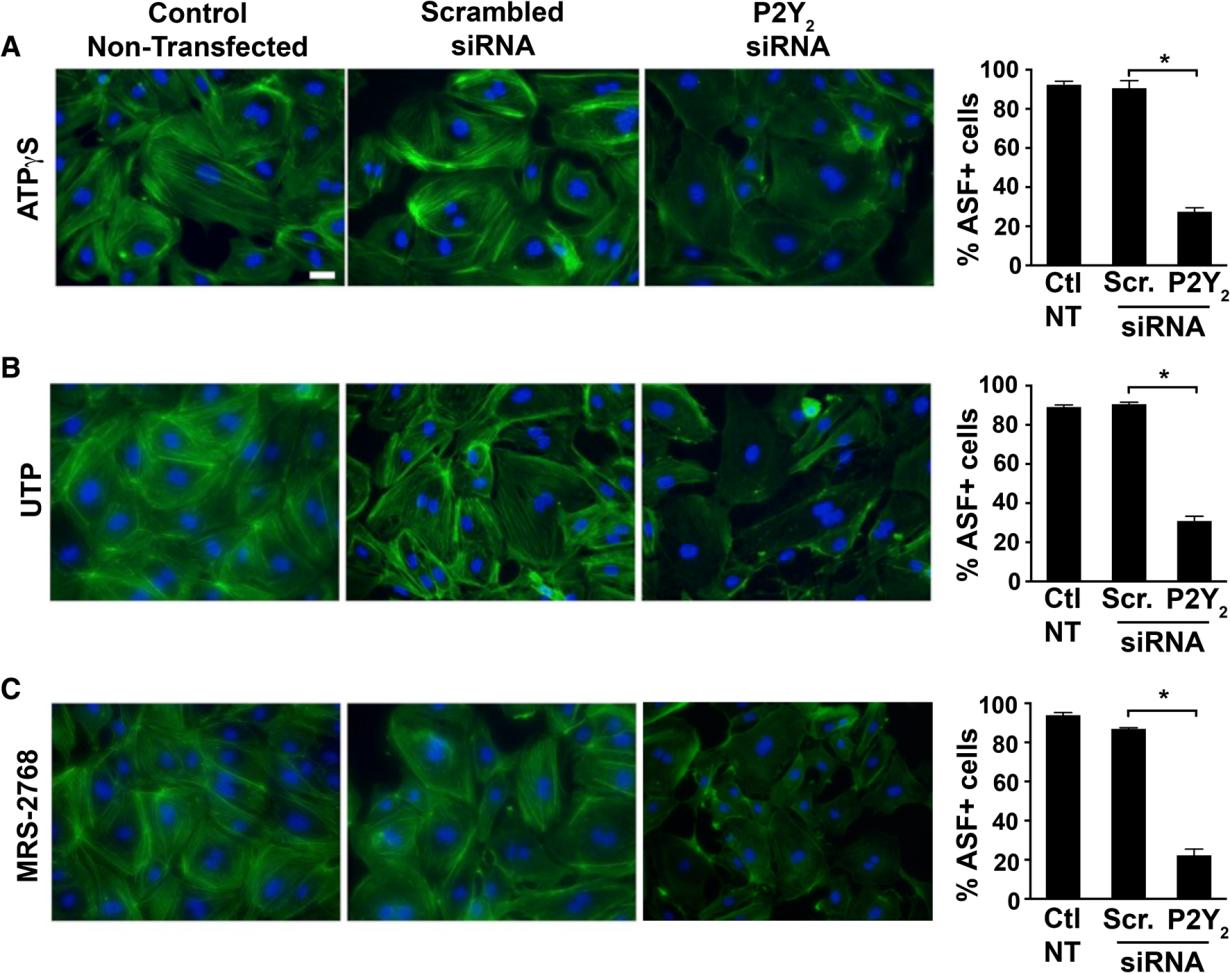
siRNA-mediated knockdown of *P2Y*_*2*_ receptor attenuated agonist-induced ASF formation. HUVECs transiently transfected with either scrambled (Scr.) or *P2Y*_*2*_-specific siRNA were exposed to 100 μmol/l ATPγS, 100 μmol/l UTP, or 10 μmol/l MRS-2768 for 1 h and fixed. Representative epifluorescence images (×40 objective) of cells stained with ActinGreen™488 and NucBlue^®^ show higher intensity staining and prominent ASF in non-transfected controls (*left panels*) and in cells transfected with scrambled siRNA (*middle panels*) compared to cells transfected with P2Y_2_ siRNA (*right panels*) upon stimulation with **a** ATPγS, **b** UTP, or **c** MRS-2768. Bar graphs indicate quantification of ASF from multiple experiments; *n* = 5 experiments; *P ≤ 0.05; *scale bar* 10 μm

We next investigated if the observed ASF formation in the endothelial cells under shear stress conditions is dependent on P2Y_2_ receptors. Phalloidin staining of cells subjected to 6 h of shear stress shows increased formation of ASF with numerous fibers running along the long axis of the cells in the direction of flow (arrowheads in Fig. 2C—d, e and magnified in f, g), which is less prominent and confined to the cell boundaries in both the static conditions (Fig. 2C—a–c) and when the receptors were knocked down (Fig. 2C—h). Similar to a previous report [16], we observe extracellular nucleotide-induced ASF formation in the HUVECs to be P2Y_2_-dependent, under static conditions (Fig. 3). Since the P2Y_2_ receptor is activated by both ATP and UTP, we used both these ligands as well as the P2Y_2_-specific agonist, MRS-2768 [27]. When compared both to the non-transfected controls and cells transfected with the scrambled siRNA, we observe greater than 60 % significant decrease in ASF formation in cells transfected with P2Y_2_ siRNA in the presence of 100 μmol/l ATPγS (Fig. 3a), 100 μmol/l UTP (Fig. 3b), and 10 μmol/l MRS-2768 (Fig. 3c).

### RGD integrin-binding domain of P2Y_2_ receptor is important for cell alignment and ASF formation

**Fig. 4:**
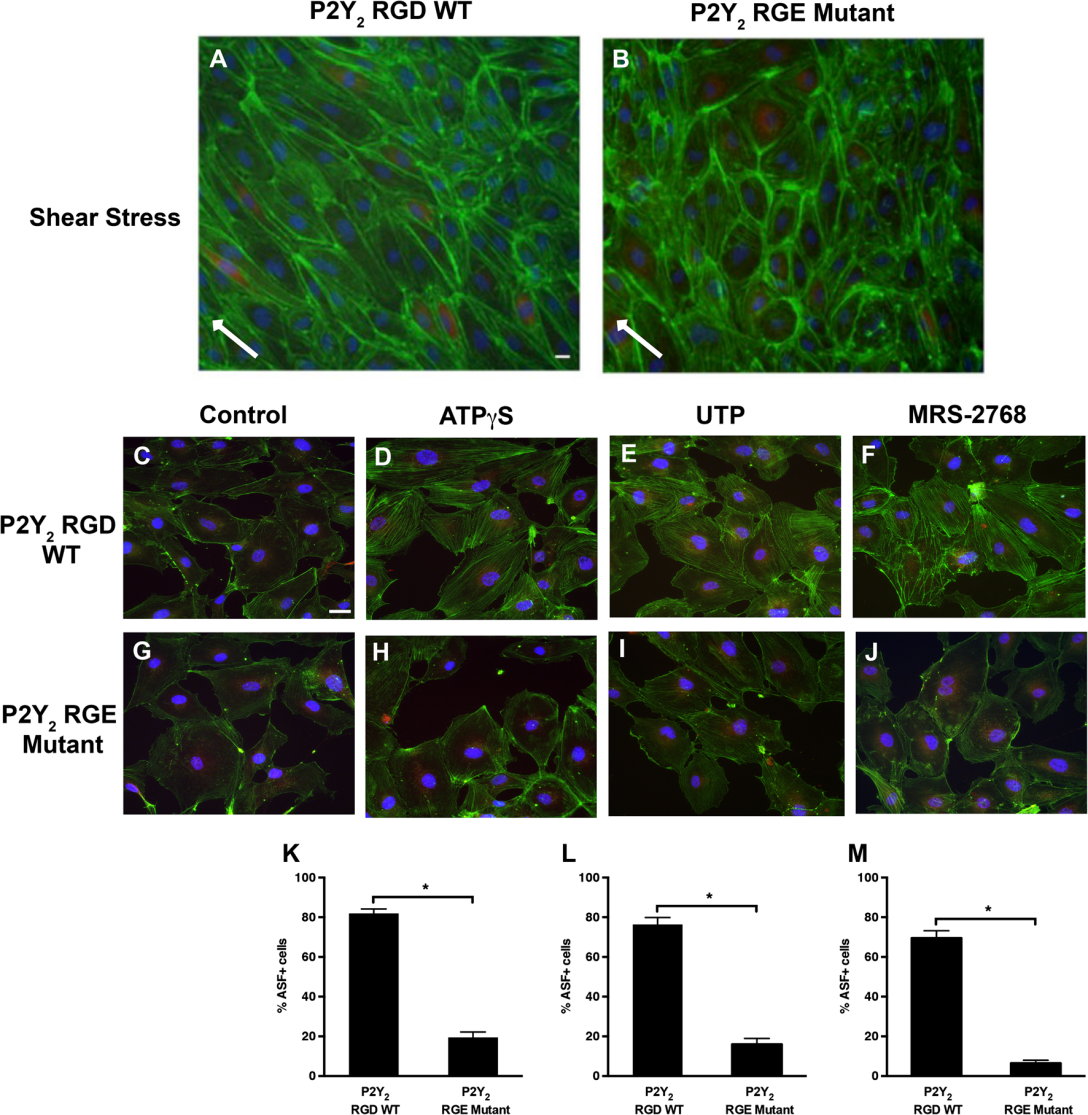
RGD-integrin binding domain of the P2Y_2_ receptor modulates shear stress-mediated cell alignment and agonist-induced ASF formation. Cells were transduced with either P2Y_2_ RGD WT (**a**, **c**–**e**) or P2Y_2_ RGE mutant (**b**, **g**–**j**) receptors and subjected to shear stress for 6 h (**a**, **b**) or receptor agonists for 1 h (**c**–**j**). Epifluorescence images (×20 objective in **a**, **b**, and ×40 objective in **c**–**j**) show representative HUVEC monolayers that were fixed and stained with anti-HA antibody (Red), ActinGreen™488, and NucBlue^®^ for HA-tagged P2Y_2_ receptor, actin, and the nucleus, respectively. Cells expressing the P2Y_2_ RGD WT receptor show cell alignment (**a**) in the direction of flow (*white arrows*) compared to cells expressing the P2Y_2_ RGE mutant receptors (**b**). Similarly, agonist-induced ASF formation is prominent in cells expressing P2Y_2_ RGD WT receptors (**d**–**f**) compared to control (**c**) or cells expressing P2Y_2_ RGE mutant receptors (**g**–**j**). Bar graphs (**k**–**m**) indicate quantification of ASF from multiple experiments; *n* = 7 experiments; *P ≤ 0.05; *scale bars* 10 μm

Fluid shear stress activates integrin signaling in endothelial cells in a Rho-GTPase-dependent manner [19, 25, 30]. We, therefore, tested if the RGD integrin-binding domain of P2Y_2_ receptors is involved in shear stress-mediated cell alignment and extracellular nucleotide-induced ASF formation in the HUVECs. Cells expressing the HA-tagged P2Y_2_ RGD WT or P2Y_2_ RGE mutant receptor were subjected to either shear stress for 6 h or exposed to ATPγS, UTP, or MRS-2768 for 1 h. The cells expressing the P2Y_2_ RGE mutant receptor show impaired cell alignment in response to shear stress compared to cells expressing the P2Y_2_ RGD WT receptors (Fig. 4a, b). When the degree of alignment was quantified, we observe 44.7 % of the cells transduced with the WT receptors to be aligned in the direction of flow compared to only 5.1 % in cells with the RGE mutant receptor (*P* = 0.000001; Online Resource 1G and H). Additionally, compared to unstimulated control cells (Fig. 4c), HUVECs expressing the P2Y_2_ RGD WT receptors show agonist-stimulated formation of ASF with prominent fibers randomly distributed throughout the cell (Fig. 4d–f). Moreover, the agonist-induced formation of ASF was negligible in unstimulated cells (Fig. 4g) and significantly decreased in about 60–63 % of the cells expressing the P2Y_2_ RGE mutant receptors under static conditions (Fig. 4h–j, k–m). Furthermore, the actin staining was observed primarily along the periphery in both the unstimulated cells and cells expressing the RGE mutant receptors. Although we did not silence the endogenous P2Y_2_ receptor expression prior to overexpressing the P2Y_2_ RGE mutant receptor, it is likely that the expression of recombinant receptors outnumbers the endogenous receptors thus creating a dominant negative effect [31, 32]. Taken together, these data suggest that P2Y_2_ receptor-integrin interacts to play a role in nucleotide-induced endothelial cell alignment and ASF formation.

### P2Y_2_ receptor mediates shear stress-regulated cytoskeletal alterations and eNOS phosphorylation in HUVECs

**Fig. 5:**
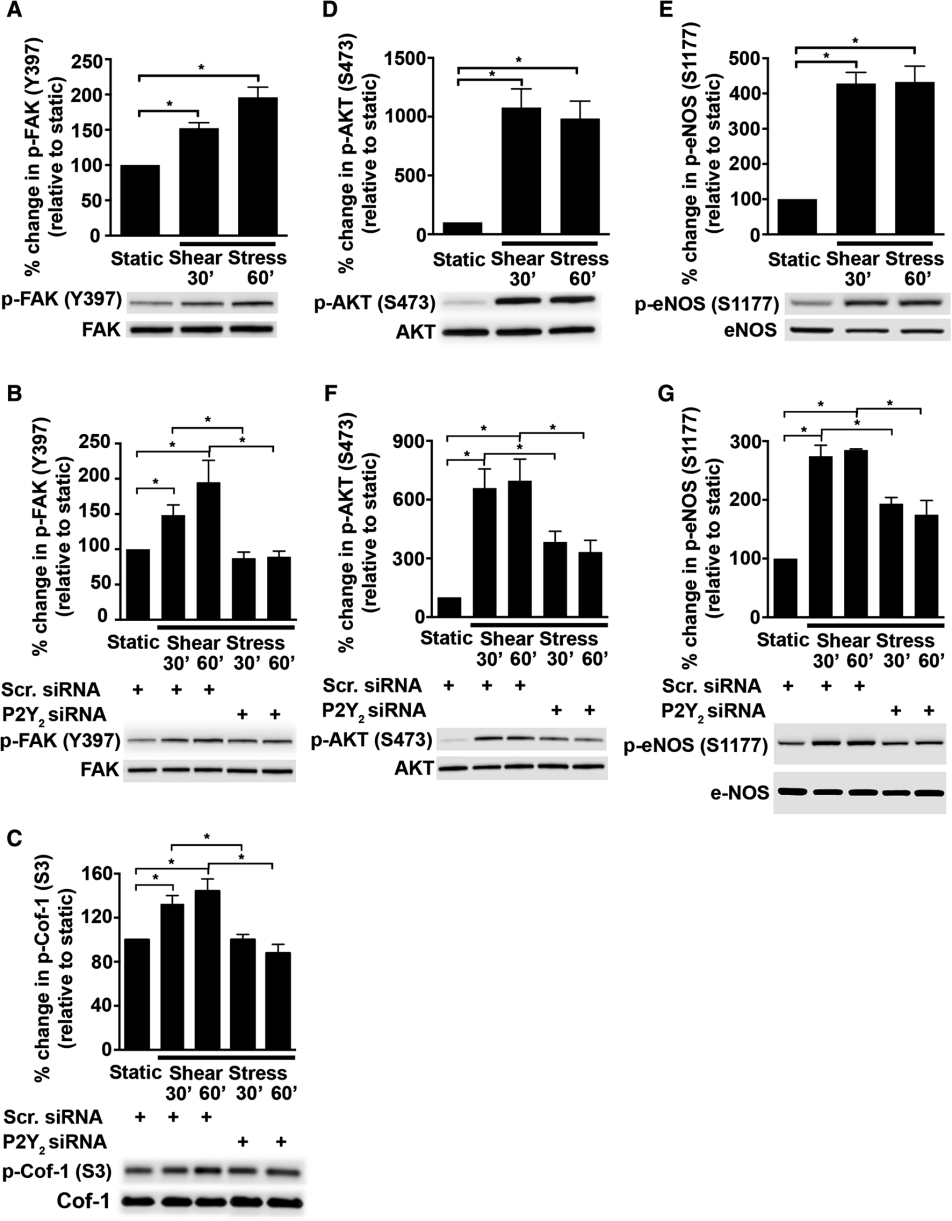
Shear stress-induced phosphorylation of FAK, Cofilin-1, AKT, and eNOS is dependent on the P2Y_2_ receptor. P2Y_2_ receptor-specific siRNA was transiently transfected into the HUVECs. The cells were subjected to shear stress or kept static for either 30 or 60 min and then lysed. Subsequently, the cell lysates were subjected to immunoblotting with the antibodies for FAK (Y397), cofilin-1 (S3), AKT (S473), and eNOS (S1177). Bar graphs with representative immunoblots show that shear stress increases phosphorylation of FAK (Y397; 125 kDa) (**a**) and that this is dependent on P2Y_2_ receptors (**b**). In addition, the phosphorylation of Cofilin-1 (Cof-1) at S3 (19 kDa) decreases upon P2Y_2_ receptor knockdown (**c**). Furthermore, shear stress (30 and 60 min) activates AKT (S473; 60 kDa) (**d**) and eNOS (S1177; 140 kDa) (**e**) and that this activation is dependent on P2Y_2_, respectively (**f**, **g**). Percent change in phosphorylation is relative to static cells (**a**, **d**, **e**) or cells transfected with the scrambled (Scr.) siRNA (**b**, **c**, **f**, **g**); *n* = 3–7 experiments; *P ≤ 0.05

FAK activation is an important component of integrin-mediated mechanosensing and motility in cells [19, 30, 33]. We therefore determined whether P2Y_2_ receptors play a role in shear stress-induced activation of FAK. We show that shear stress indeed increases FAK phosphorylation at Y397 at both 30 min (Fig. 5a; 1.52 ± 0.08-fold; *P* = 0.002) and 60 min (Fig. 5a; 1.96 ± 0.14-fold; *P* < 0.0001) when compared to static control. Knockdown of the P2Y_2_ receptor decreases shear stress-induced phosphorylation of FAK at both time points analyzed (Fig. 5b; 30 min: 41.1 ± 5.8 %; *P* = 0.009; 60 min: 54.1 ± 4 %; *P* = 0.0003). An important function of FAK is to transduce the mechanosignals that influence cell structure in response to shear stress [33–36]. To address the molecular events that bridge P2Y_2_ receptors to endothelial cell adaptation to flow, we focused on signaling molecules influenced by FAK that regulate endothelial cytoskeletal architecture. In view of this, we examined the activation status of cofilin-1, a downstream target of FAK and a regulator of actin polymerization. In Fig. 5c, we show that knockdown of the P2Y_2_ receptor expression in HUVECs reduced phosphorylation of cofilin-1 at 30 min (24.4 ± 3.5 %; *P* = 0.02) and 60 min of shear stress (39 ± 5.5 %; *P* = 0.0001), similar to FAK.

Shear stress and nucleotide activation of the P2Y_2_ receptor also results in FAK-mediated phosphorylation of AKT and eNOS [26, 34]. Concurrent with these studies, we find shear stress induces the phosphorylation of AKT at S473 (Fig. 5d; 30 min: 10.8 ± 1.6-fold; *P* < 0.0001 and 60 min: 9.9 ± 1.5-fold; *P* = 0.0003) and eNOS at S1177 (Fig. 5e; 30 min: 4.3 ± 0.3-fold; *P* < 0.0001 and 60 min: 4.3 ± 0.5-fold; *P* < 0.0001). Furthermore, knockdown of the P2Y_2_ receptor in HUVECs has an inhibitory effect on shear stress-induced phosphorylation of AKT (Fig. 5f; 30 min: 41.9 ± 9.1 %; *P* = 0.048, and 60 min: 52.3 ± 8.6 %; *P* = 0.02) and eNOS (Fig. 5g; 30 min: 29.5 ± 3.9 %; *P* = 0.001, and 60 min: 38.6 ± 8.6 %; *P* = 0.004).

**Fig. 6:**
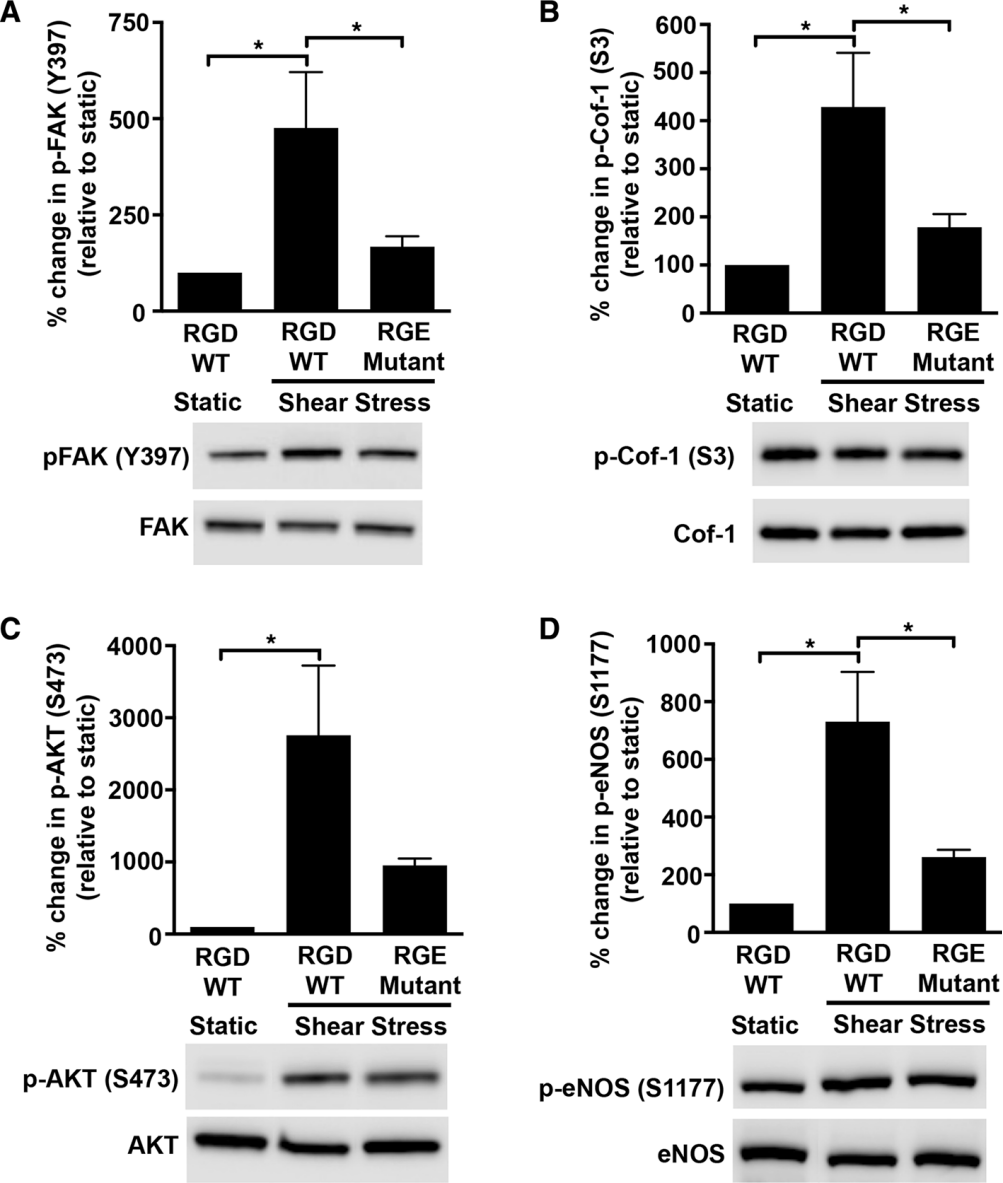
P2Y_2_ receptor-integrin interaction is important for shear stress-induced activation of FAK, cofilin-1, AKT, and eNOS. HUVECs transduced with either P2Y_2_ RGD WT or P2Y_2_ RGE mutant receptor constructs were subjected to shear stress or kept static for 30 min and then lysed. Cell lysates were analyzed by immunoblotting with antibodies to **a** FAK (Y397), **b** Cofilin-1 (S3), **c** AKT (S473), and **d** eNOS (S1177). Graphs with representative immunoblots show that shear stress-induced phosphorylation of FAK (Y397; 125 kDa), Cofilin-1 (S3; 19 kDa), AKT (S473; 60 kDa), and eNOS (S1177; 140 kDa) are dependent on the RGD integrin-binding domain of P2Y_2_ receptors. Percent change in phosphorylation is relative to static cells expressing the P2Y_2_ RGD WT receptor constructs; *n* = 5–6 experiments; *P ≤ 0.05

Since FAK is an important component of integrin signaling, we tested if the RGD domain of the P2Y_2_ receptor that is known to interact with the integrins was required for the shear stress response. Indeed, HUVECs expressing the P2Y_2_ RGE mutant receptors show reduced levels of phosphorylated FAK (Fig. 6a; 64.9 ± 5.7 %; *P* = 0.04) and phosphorylated cofilin-1 (Fig. 6b; 58.4 ± 6.4 %; *P* = 0.04). Interestingly, we also find that the P2Y_2_ RGE mutant receptor has a similar inhibitory effect on the activated states of both AKT (Fig. 6c; 65.5 ± 3.4 %; *P* = 0.07) and eNOS (Fig. 6d; 64.3 ± 3.5 %; *P* = 0.01) suggesting that P2Y_2_ receptor-integrin interactions play a role in these shear stress-mediated cell alignment and vasodilatory responses.

### P2Y_2_ receptors play a role in shear stress-mediated wound closure

**Fig. 7:**
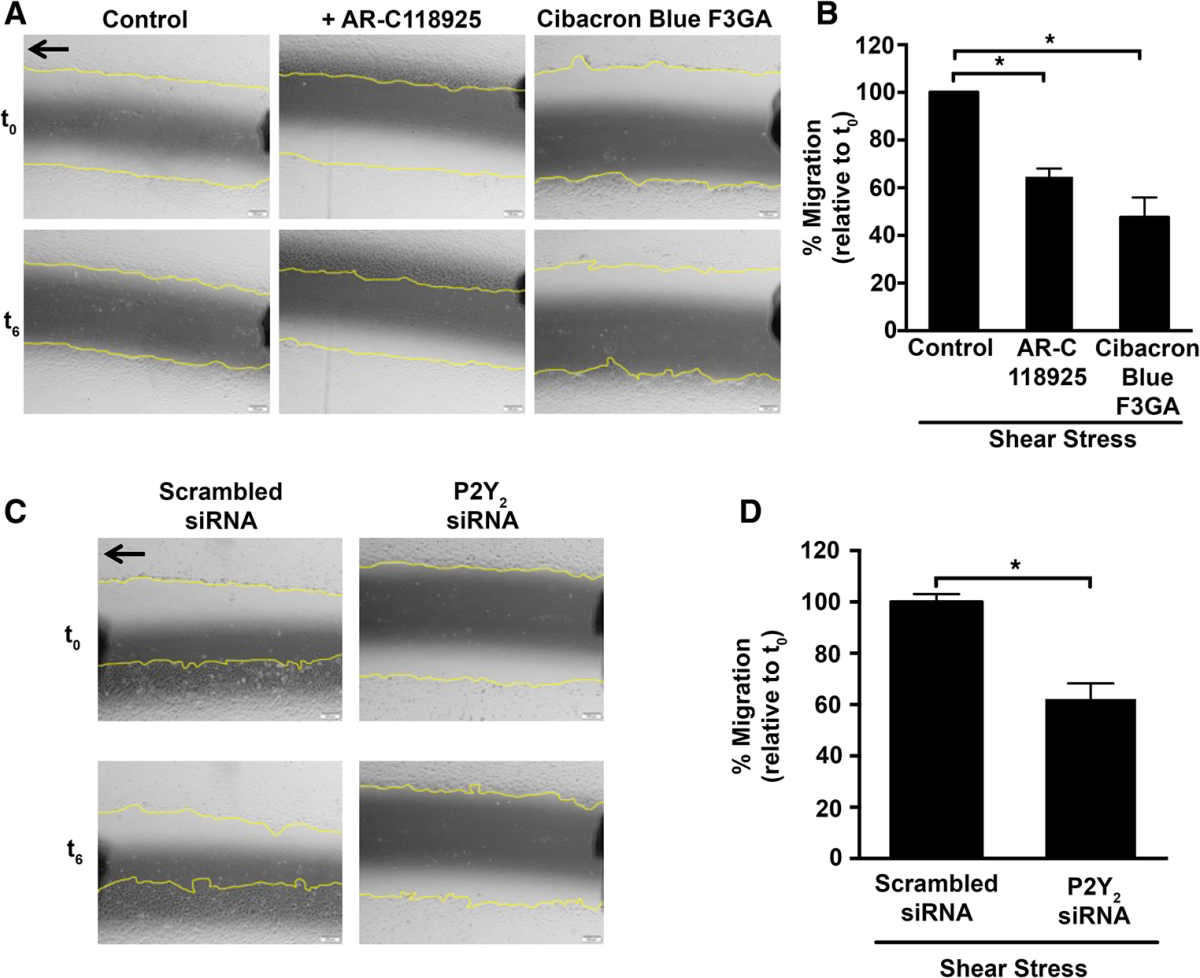
Both pharmacological inhibition and siRNA-mediated knockdown of the P2Y_2_ receptor attenuate shear stress-mediated wound closure. The in vitro scratch wound closure was assayed in **a**, **b** cells exposed to P2Y_2_ receptor antagonists, and **c**, **d** cells transfected with either scrambled or *P2Y*_*2*_-specific siRNA. HUVEC monolayers were wounded in the direction of flow (*black arrow*) and subjected to shear stress for 6 h. Cells were imaged (**a**, **c**) at the same position with representative phase contrast images (×10 objective) acquired at *t*_0_ (0 h) indicating the initial wound and at *t*_6_ (6 h after shear stress). Bar graphs show quantification of these images with slower wound closure in the presence of **b** P2Y_2_ receptor antagonists or **d** *P2Y*_*2*_-specific siRNA; *n* = 5 independent experiments; *P ≤ 0.05

A previous study has shown that ATP and UTP stimulate FAK phosphorylation in HUVECs suggesting an important role for P2Y_2_ receptors in cell spreading and migration [16]. Using our in vitro shear stress model, we evaluated the role of P2Y_2_ receptors in wounded HUVEC cultures. Consistent with prior studies, we observe enhanced wound closure under shear stress compared to static (data not shown). Furthermore, we observe a 35.3 ± 3.4 and 52.4 ± 8.3 % inhibition in wound closure with AR-C118925 (*P* = 0.0002) and Cibacron Blue F3GA (*P* < 0.0001), respectively (Fig. 7a, b). A similar impairment in wound closure is observed with siRNA knockdown of the P2Y_2_ receptors and is consistent with the effect of the receptor antagonists (Fig. 7c, d; 37.8 ± 6.1 %; *P* = 0.002).

## Discussion

The endothelial cells lining the vessel wall have the exquisite ability to adapt to hemodynamic forces in response to blood flow. Several purinergic mechanisms have been described in the context of vascular physiology, although the role of P2Y_2_ receptors in regulating cytoskeletal alterations under shear stress in endothelial cells has so far remained unclear. HUVECs express P2X as well as P2Y receptors, and are shown to have high ATP signaling activity on their cell surface [37–40]. Previously, we reported that shear stress upregulated the P2Y_2_ receptor in HUVECs [12, 41] and recent studies from others have suggested that these receptors mediate mechanotransduction [14, 15, 26]. Our primary findings in the present study are that P2Y_2_ receptors modulate endothelial cell alignment, ASF formation, and cell migration in HUVECs and we thereby propose a novel role for P2Y_2_ receptors in shear stress-induced endothelial cell remodeling.

Endothelial cells adapt to shear stress by aligning and migrating in the direction of flow. Changes in endothelial cell shape and cytoskeletal organization are among the most rapid adaptations induced by blood flow, failure of which contributes to endothelial damage, atherogenesis, and abnormal repair. The endothelial cells in large blood vessels exposed to laminar flow patterns are well aligned compared to those at branch points that are susceptible to atherosclerosis [3]. In our study, we observe that static HUVECs display a polygonal cobblestone-like morphology that transitions to a spindle-shaped monolayer aligning in the direction of flow under shear stress conditions, which is comparable to the in vivo endothelial cell morphology. Concurrent with endothelial cell alignment in the direction of flow, ASF formation determines endothelial cell stiffness and contributes to the resistance against fluid shear [19, 30, 42–45]. In fact, the HUVECs when subjected to shear stress not only aligned but also assembled stress fibers in the direction of flow. In contrast, both the antagonism and the knockdown of the P2Y_2_ receptor resulted in less prominent alignment as well as attenuation of ASF formation. Exposure of a confluent endothelial monolayer to shear stress results in chemomechanical signaling-induced increases in intracellular tensions along the direction of flow [46] accompanied by reorganization of cell junction-associated proteins [47]. Indeed, Liao et al., have demonstrated that UTP activation of P2Y_2_ receptors in human coronary aortic endothelial cells caused a transient translocation of the P2Y_2_ receptor to cell–cell junctional zones. They also demonstrated that the P2Y_2_ receptor transiently interacted with VE-Cadherin, a protein specific to endothelial adherens junction, and activated Rac-1 in a Src- and VEGFR2-dependent manner [48]. Moreover, there is evidence that endothelial cell responses to shear stress are mediated in part by a mechanosensory complex consisting of VE-cadherin, PECAM-1, VEGFR2 [49], and Rac-1, which is an integral component of endothelial cell–cell contact remodeling [50]. Therefore, it is likely that in our study the inactivation of P2Y_2_ receptors could have an effect on the adherens junctions. Further, we observed exogenous nucleotides to induce prominent ASF formation in static HUVEC cultures in a P2Y_2_-dependent manner similar to the cells under shear stress. While several groups have shown shear stress-induced ATP release to promote P2 receptor-mediated increases in the concentration of intracellular free calcium and vasodilators in endothelial cells [9, 51, 52], a previous study reported exogenous nucleotides increase formation of ASF in static HUVEC cultures [16]. Therefore, it is possible that the extracellular ATP released during blood flow modulates cytoskeletal alterations via P2Y_2_ receptor activation, thus pointing to the relevance of these receptors in vascular remodeling downstream of mechanotransduction. These findings are the first indication that the P2Y_2_ receptor plays a role in shear stress-mediated endothelial cell alignment and ASF formation.

Given that many signaling molecules are associated with the cytoskeleton, it is possible that the shear stress-induced intracellular tension results in coupling the mechanical stimuli to chemical responses in cells. Studies have demonstrated that mechanotransduction in endothelial cells in response to shear stress not only activates integrins but also enhances their avidity and affinity for interaction with other proteins [30, 43, 49]. Here, we present evidence that the RGD integrin-binding domain of P2Y_2_ receptors influences both HUVEC alignment and ASF formation in response to shear stress. The interaction of P2Y_2_ receptors with integrins could possibly transduce mechanical stimuli into chemical cues, thereby altering the cytoskeleton. In fact, Erb et al. showed that P2Y_2_ receptors associate with integrins via the RGD domain of the P2Y_2_ receptor and that this interaction is lost when the domain is mutated to RGE [22]. It is therefore likely that a similar kind of interaction is plausible in the current model.

Several studies in endothelial cells have shown FAK to be a key second messenger in mechanotransduction [34, 35] and that activation of FAK (phosphorylation at Tyr397) in response to extracellular nucleotides induces cytoskeletal alterations and directional migration in endothelial cells [25, 53]. In this study, we show for the first time those P2Y_2_ receptors mediate shear stress-induced FAK activation in HUVECs and that this is dependent on the RGD integrin-binding domain of the P2Y_2_ receptor. Actin polymerization and de-polymerization is an active event in response to shear stress that is important for the formation of ASF and cytoskeletal remodeling [14]. One of the important regulators of actin polymerization is cofilin-1, which in its phosphorylated state enables actin polymerization and ASF formation [14, 17, 54, 55]. Further evidence supporting a role for the P2Y_2_ receptor in regulating actin cytoskeletal dynamics comes from our observation of reduced cofilin-1 phosphorylation either after *P2Y*_*2*_ knockdown or in the presence of mutant P2Y_2_ receptors lacking the RGD integrin-binding motif. The rapid reorganization of the cytoskeleton and downstream signaling during endothelial cell alignment in response to flow predominantly involves sequential and coordinated activation of the small GTPases [17, 21, 22, 25, 30, 36]. Although we did not measure the activity of the small GTPases, per se, the observed flow-induced cell alignment and ASF formation in our model of shear stress suggest that P2Y_2_ receptor activation by extracellular nucleotides and its association with integrins modulates the regulation of small GTPases. This is supported by studies showing that stimulation of P2Y_2_ receptors by UTP activates small GTPases in an integrin-dependent manner as observed in astrocytoma cells as well as in human coronary artery endothelial cells [17, 21].

In addition to shear stress-mediated cytoskeletal alterations, endothelial cells release vasoactive substances including NO. Recently Wang et al., have shown in endothelium-specific *P2Y*_*2*_ knockout mice that these receptors induce phosphorylation of AKT as well as eNOS via the activation of heterotrimeric G proteins, G_q_ and G_11,_ thereby regulating vascular tone and blood pressure [26]. In line with these findings, we observed activation of AKT and eNOS in the HUVECs in a P2Y_2_ receptor-dependent manner. Although, the observation of reduced shear stress-mediated AKT activation did not reach statistical significance in cells expressing the P2Y_2_ RGE mutant receptors, we found a significant decrease in eNOS activation, as compared to cells expressing the P2Y_2_ RGD WT receptor. As previously described, this could be attributed to the fact that the P2Y_2_ RGE mutant receptor requires a much higher agonist concentration for AKT activation [21]. In addition, FAK is known to be central to flow-induced dilation in coronary arterioles via regulation of AKT and eNOS activities [34] while shear stress and extracellular nucleotides activate AKT via the stimulation of integrins and PI3 K [16, 49, 56]. Furthermore, previous studies from our group and others have shown that ATP release in response to shear stress in endothelial cells regulates NOS3 expression and vasodilation via P2X4 receptors [12, 13]. Collectively, these studies and our in vitro data provide supportive evidence that flow-induced eNOS activity is regulated both by the mechanotransducing function of endothelial P2Y_2_ receptors and by ionotropic P2X4 receptors.

An intact endothelium is important for maintaining vascular homeostasis and preventing pathological conditions. Sustained exposure to flow not only induces cytoskeletal remodeling but also is necessary for timely repair of the injured endothelium. In our study, pharmacological inhibition and siRNA knockdown of P2Y_2_ receptors impaired the shear stress-driven wound closure in a wounded HUVEC monolayer. Reports have suggested that both the P1 adenosine and the P2Y receptors mediate trophic effects on cultured endothelial cells [57–61]. Furthermore, it has been demonstrated that nanomolar concentrations of ATP are sufficient to have a proliferative effect in HUVECs via the formation of an ATP-VEGF-A_165_ complex [62]. Nevertheless, the effect on wound closure that we observed in this study was as early as 6 h, which is consistent with a prior study that relates this early shear stress response primarily to cell migration rather than proliferation [63]. Reports show extracellular nucleotides to induce endothelial cell responses such as FAK activation and PI3K-dependent migration [16]. Additionally, shear stress is known to mediate endothelial cell alignment by integrin activation via stimulation of PI3K [49]. Given that downstream integrin signaling events include activation of FAK, PI3K, and AKT, there is reason to believe that the P2Y_2_ receptor-integrin interaction plays a role in shear stress-induced vasodilatory responses as well as in adaptive responses including cell alignment and migration.

## Conclusion

**Fig. 8:**
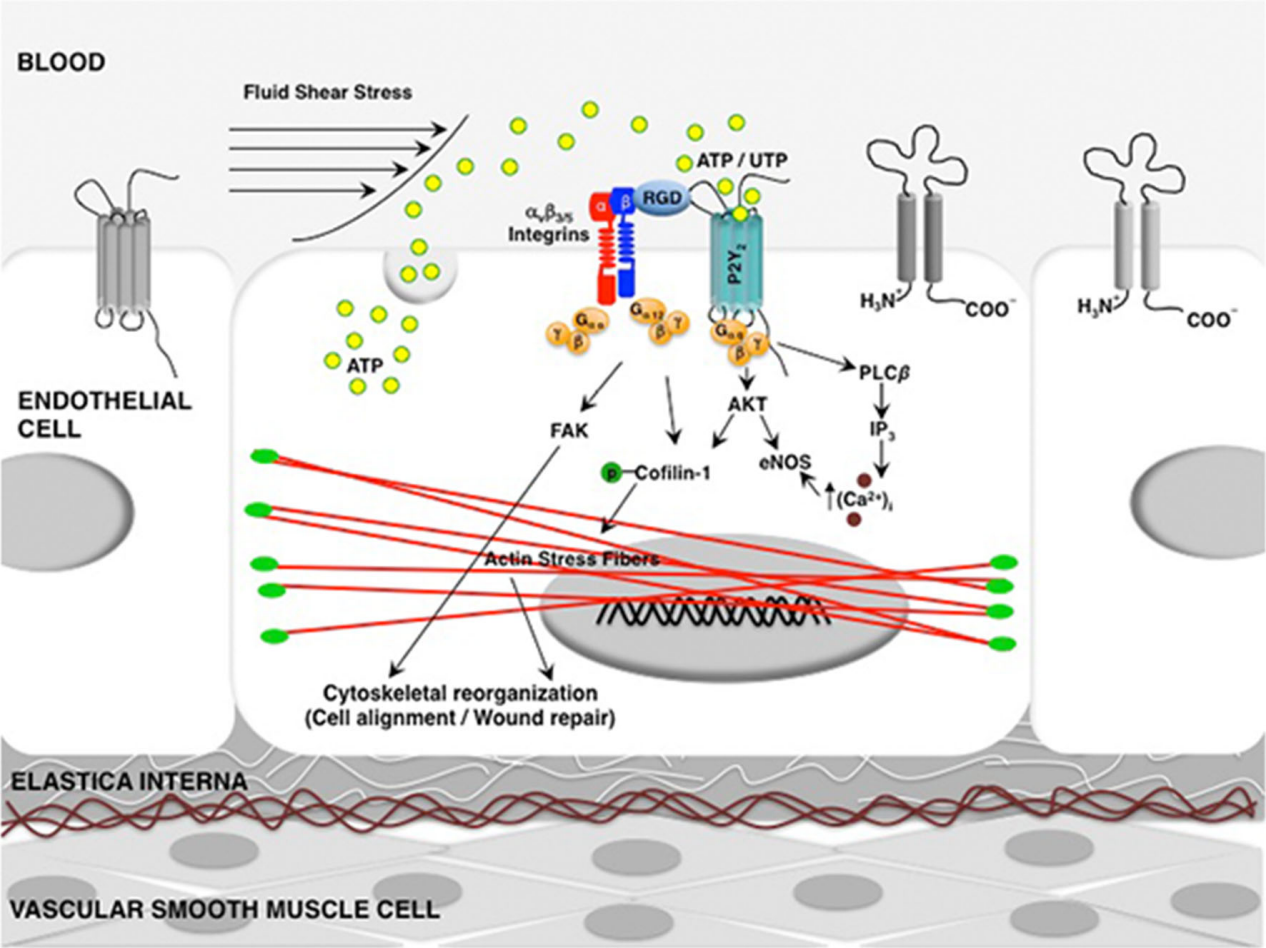
A schematic of the role of the P2Y_2_ receptor in shear stress-mediated cell alignment, formation of ASF and wound closure. It is well established that extracellular nucleotides activate P2Y_2_ receptors and increase the intracellular calcium concentration via Gαq, phospholipase C (PLC)-β, and inositol triphosphate (IP_3_) [66, 67]. In this study, under shear stress conditions, we show P2Y_2_ receptors (1) phosphorylate AKT and eNOS; (2) modulate shear stress-induced endothelial cytoskeletal alterations via their integrin-binding RGD domain through the activation of FAK and cofilin-1; and (3) promote wound repair in HUVECs. Additionally, in support of this model, previous studies have demonstrated that activation of AKT regulates eNOS [68] and cofilin-1 activities [69]

In summary, our findings emphasize the role of P2Y_2_ receptors not only in modulating endothelial cell alignment and ASF formation but also in enhancing wound closure (Fig. 8). Thus, these receptors cannot be ruled out as an important part of the mechanotransduction complex. Although the HUVECs are not representative of all blood vessels, they have been extensively used as an in vitro model to study vascular remodeling and endothelial cell function. Further, the hemodynamic forces are important modulators of vascular remodeling not only in arteries but also in veins. Support for this comes from studies that show exposure of the vein to arterial flow results in its arterialization accompanied by adaptive responses, including gene transcription and structural remodeling of venous bypass grafts [64, 65]. The shear stress-induced cytoskeletal alterations in endothelial cells may have common biochemical signaling pathways but to our knowledge this is the first study demonstrating modulation of shear stress-induced cytoskeletal alterations by P2Y_2_ receptors in HUVECs. However, a better understanding of the consequences of the interactions between shear stress and the purinergic signaling network can influence the success of therapeutic modalities in treating cardiovascular disease progression and clinical outcomes.

## Electronic supplementary material

Below is the link to the electronic supplementary material.

Supplementary material 1 (PDF 475 kb)

## Abbreviations

HUVECs: Human umbilical vein endothelial cells
IVF: In vitro fertilization
Pa: Pascal
ASF: Actin stress fibers
FAK: Focal adhesion kinase
RGD: Arg-Gly-Asp
RGE: Arg-Gly-Glu
NOS: Nitric oxide synthase
