# Supplementary material for: P2Y2 receptor modulates shear stress-induced cell alignment and actin stress fibers in human umbilical vein endothelial cells
Source: Cell Mol Life Sci. 2016 Sep 20;74(4):731–46. doi: 10.1007/s00018-016-2365-0 (PMC5272905; doi:10.1007/s00018-016-2365-0)

$P2Y_2$ Receptor Modulates Shear Stress-Induced Cell Alignment and Actin Stress Fibers in Human Umbilical Vein Endothelial Cells

Ramasri Sathanoori[1], Paulina Bryl-Gorecka[1], Christa E. Müller[2], Laurie Erb[3], Gary A. Weisman[3], Björn Olde[1], and David Erlinge[1]*

[1]Department of Cardiology, Clinical Sciences, Lund University, Sweden

[2]PharmaCenter, Pharmaceutical Institute, Pharmaceutical Chemistry I, University of Bonn, Germany

[3]Department of Biochemistry, University of Missouri, Columbia, USA

Journal Name: Cell and Molecular Life Sciences

[*]Corresponding Author:

Ramasri Sathanoori

Email: Ramasri.Sathanoori@med.lu.se

Online Resource 1 Quantification of shear stress-induced cell alignment

Frequency distribution graphs representing the degree of cell orientation: (A) under shear stress and (B) in static; (C) in the absence and (D) presence of receptor antagonists; (E) in cells transfected with scrambled and (F) *$P2Y_2$* receptor-specific siRNA; and (G) in cells expressing the $P2Y_2$ RGD WT receptors and (H) $P2Y_2$ RGE mutant receptors. The maximal percentage of cells aligned at 45° angle (direction of flow) in all of the controls under shear stress (A, C, E, G) compared to the random orientation observed in the experimental groups (B, D, F, H). The asterisk represents statistical significance of percent cells aligned in the direction of flow ($*P \leq 0.05$).

Online Resource 2 Validation of knockdown $P2Y_2$ receptor protein expression

Representative immunoblots (A) probed with the anti-$P2Y_2$ and anti-HA antibodies. The 42 kDa band corresponding to $P2Y_2$ was confirmed using anti-HA antibodies in lysates from HUVECs overexpressing the HA-tagged $P2Y_2$ RGD WT receptors. (B) Representative immunoblots and bar graph show shear stress to increase $P2Y_2$ protein in cells transfected with scrambled (Scr.) siRNA relative to the static Scr. siRNA control ($\#P \leq 0.05$). Under shear stress conditions, the HUVECs show decreased receptor expression in the presence of *$P2Y_2$*-specific siRNA ($*P \leq 0.05$). n = 4 experiments

Online Resource 1

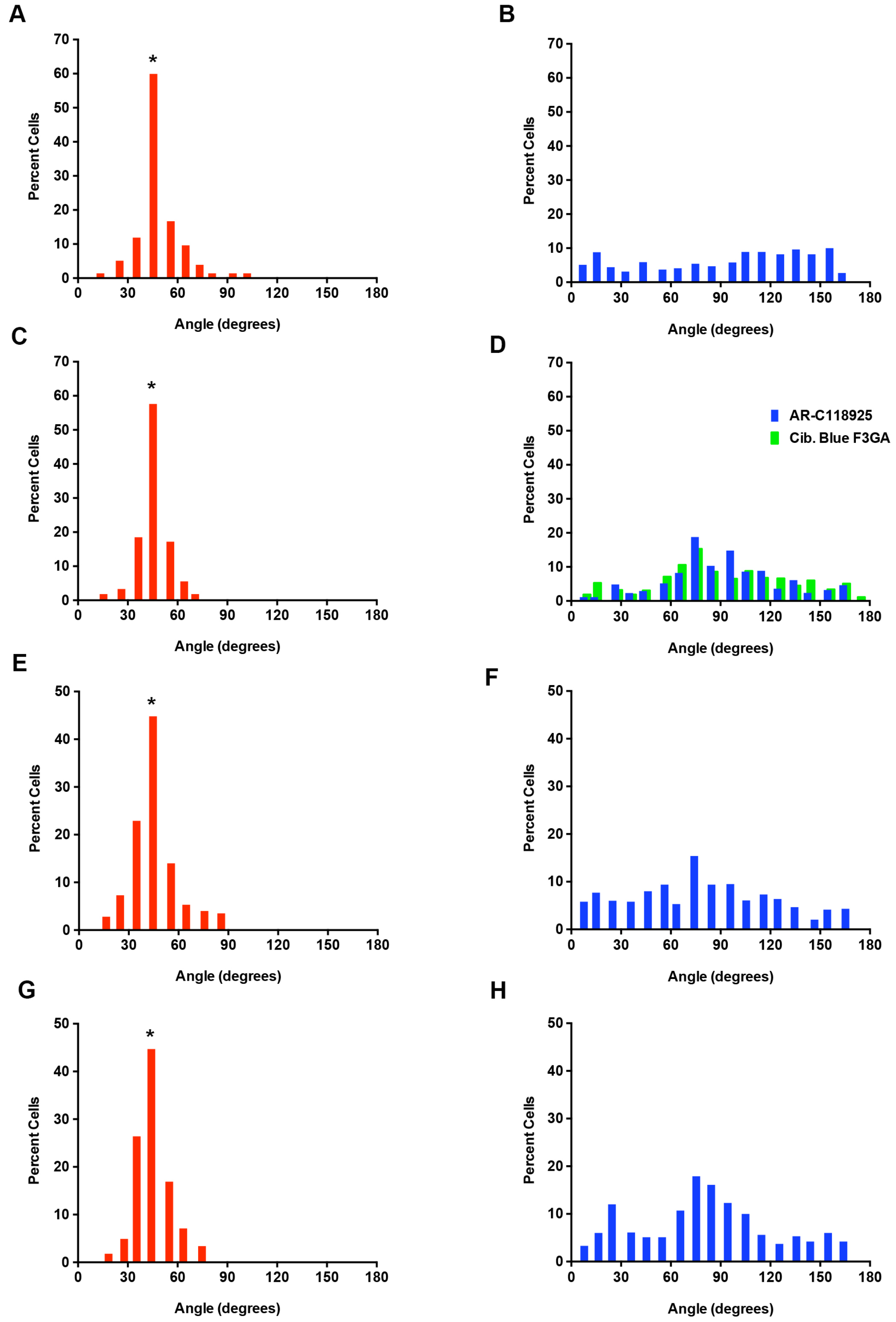

Online Resource 2

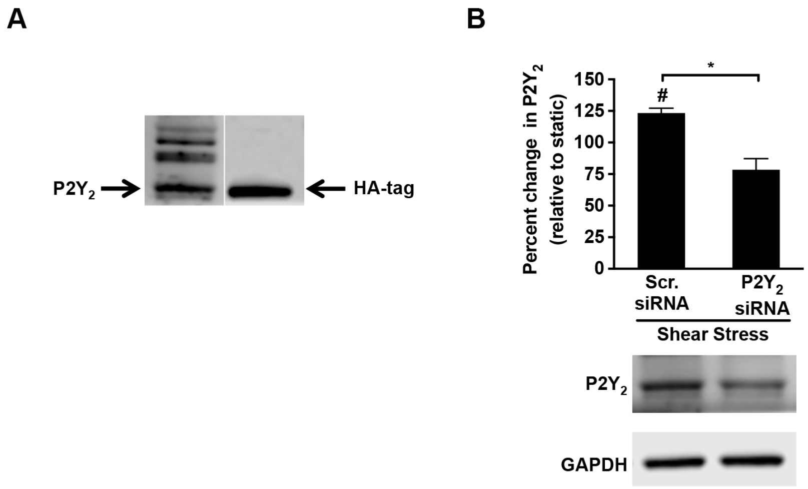

Supplement: Supplementary file 1 — Supplementary material 1 (PDF 475 kb) [file 18_2016_2365_MOESM1_ESM.pdf]
